# Age impacts left atrial functional remodeling in athletes

**DOI:** 10.1371/journal.pone.0271628

**Published:** 2022-07-15

**Authors:** Cynthia Cousergue, Eric Saloux, Emmanuel Reboursière, Amélia Rocamora, Paul Milliez, Hervé Normand, Amir Hodzic

**Affiliations:** 1 Department of Cardiology, Normandie Univ, UNICAEN, CHU Caen Normandie, Caen, France; 2 EA4650 (SEILIRM), FHU REMOD-VHF, Caen, France; 3 Normandie Univ, UNICAEN, Caen, France; 4 Department of Sports Medecine, Normandie Univ, UNICAEN, CHU Caen Normandie, Inserm Comete, GIP Cyceron, Caen, France; 5 Centre de Recherche Clinique (CRC), CHU Caen Normandie, Caen, France; 6 Department of Clinical Physiology, Normandie Univ, UNICAEN, CHU Caen Normandie, Inserm Comete, GIP Cyceron, Caen, France; Policlinico Casilino, ITALY

## Abstract

**Aim:**

Age-associated changes in cardiac filling and function are well known in the general population. Yet, the effect of aging on left atrial (LA) function, and its interaction with left ventricular (LV) adaptation, remain less described when combined with high-intensity chronic training. We aimed to analyze the effects of aging on LA and LV functions in trained athletes.

**Methods and results:**

Ninety-five healthy highly-trained athletes referred for resting echocardiography were included. Two groups of athletes were retrospectively defined based on age: young athletes aged <35 years (n = 54), and master athletes aged ≥35 years (n = 41). All subjects were questioned about their sports practice. Echocardiographic analysis of LV systolic and diastolic functions (2D-echo, 3D-echo, and Doppler), as well as LA 2D dimensions and phasic deformations assessed by speckle tracking, were analyzed. Master athletes (mean age = 46.3 ± 8.3 years, mean duration of sustained training = 13.7 ± 8.9 years) exhibited significantly stiffer LV and LA with reduced LV early diastolic functional parameters (ratio E/A, peak e’, and ratio e’/a’), LA reservoir and conduit strain, whereas LA volume, LA contractile strain and LV peak a’ were higher, compared to young athletes. Multivariate regression analysis confirmed that age was predictive of peak e’, LA reservoir strain and LA conduit strain, independently of training variables. LA phasic strains were strongly associated with LV diastolic function.

**Conclusions:**

Regardless of chronic sports practice, master athletes exhibited age-related changes in LA function closely coupled to LV diastolic properties, which led to LV filling shifts to late diastole.

## Introduction

Physiological cardiac adaptation with sustained training is responsible for ventricular and atrial morphological and functional changes [1–3]. Left atrial (LA) remodeling plays an important part in the diastolic adjustment in trained athletes [4]. A close association between LA dilatation and left ventricular (LV) enlargement has been established as the consequence of a global cardiac response to the increased preload induced by chronic exercise [5]. Although LA adaptation to exercise conditioning has been related to a significant diastolic improvement [6], exercise-induced atrial remodeling can overlap the atrial size observed in cardiac diseases in which atrial dilatation is associated with higher risks of atrial fibrillation [7] and cardiovascular events [8]. A comprehensive evaluation of LA morphological and functional changes that may occur in trained athletes is required and should not be restricted to the atrial size [9]. Previous observations that have reported reference ranges for normal LA phasic strain in cohorts of trained athletes did not describe aging consequences on the variability of the LA’s response when exposed to intensive chronic exercise [10]. Age-related decline in LA function has been well described in healthy nonathletic populations and linked to LV diastolic changes [11], however, aging effects on the atrioventricular mechanical coupling have not been fully addressed in athletes.

This study aimed to analyze the impact of healthy aging on LV and LA morphological and functional remodeling using echocardiography in a cohort of highly trained young athletes and master athletes.

## Methods

### Study design

We retrospectively retrieved from our research echo-imaging database at the Hospital of the University of Caen Normandy all the echocardiograms performed for preparticipation cardiovascular screening including physical exam, ECG and transthoracic echocardiography in asymptomatic highly trained competitive athletes (≥ 6 hours of sustained training per week on a regular basis for at least 12 months) aged ≥ 18 years old, who consented to participate in the INFINITE study (Clinicaltrial.gouv NCT 03076788). The study was approved by the local research ethics committee. A single exhaustive medical form was filled for each participant. Age, gender, weight, height, artery blood pressure (BP), and heart rate (HR) were recorded alongside a complete anamnesis searching for personal and familial cardiac and systemic diseases, cardiovascular risk factors, treatments and doping, and cardiovascular symptoms. Participants were excluded if they exhibited any cardiovascular or chronic disease, cardiovascular risk factors including hypertension, dyslipidemia, and diabetes, or presented anomalies during the cardiovascular examination. Heart rate and blood pressure were measured after 10 minutes of quiet rest in a supine position. Blood pressure was averaged on duplicate successive measurements assessed using an automated monitor with an appropriate-sized arm cuff. Body mass index (BMI) was calculated as follows: [weight (kg)/height (m)^2^]. Body surface area was calculated with the Mosteller formula [12]. The subjects were questioned about their main sport discipline, the average hours/week of training during the past year, and the number of years of sustained chronic training. Two groups of athletes were compared based on age, the group of younger athletes aged under 35 years old and the group of master athletes aged ≥ 35 years old.

### Echocardiographic analysis

The echocardiographic assessment of left ventricular and atrial functions was carried out according to the current guidelines [13], using a commercially available echocardiography system (Epiq 7 equipped with an X5-1 xMATRIX-array transducer, Philips). All data were stored digitally and offline data analysis was performed blinded to the demographic and sports data (TOMTEC-Arena TTA2, TOMTEC Imaging Systems GMBH, Germany). LV dimensions were assessed from M-mode via the parasternal long-axis view. LV 3D volumes, ejection fraction (EF), and mass were obtained using the TOMTEC 4D LV analysis software. LV global longitudinal strain (GLS) was based on 3D speckle tracking. LV diastolic function was analyzed by measuring peak early (E) and late (A) transmitral velocities by pulsed-wave Doppler and peak mitral averaged (septal and lateral) annulus early (e’) and late (a’) velocities by tissue Doppler imaging. All Doppler parameters were obtained as the average value of three consecutive cardiac cycles during a brief apnoea. LV stiffness was calculated using the E/e’ and LV end-diastolic volume ratio as previously described [14].

Left atrial (LA) volume was estimated by the Biplane method of disks at end-systole from apical 4-chamber and 2-chamber views. Volumetric indexes of LA global and phasic function were assessed by calculating maximal LA volume at end ventricular systole (LAmax), minimal LA volume at end ventricular diastole (LAmin), and LA volume at pre atrial phase (LApre-A): LA total ejection fraction (%) = (LAmax-LAmin)/LAmax; LA expansion index = (LAmax-LAmin)/LAmin; LA passive ejection fraction (%) = (LAmax-LApreA)/LAmax; and LA active ejection fraction (%) = (LApreA-LAmin)/LApreA. 2D-speckle tracking measurements of LA phasic strains were performed according to current practice recommendations [15], LA-reservoir (LASr), -conduit (LAScd), and -contractile (LASct) strain were calculated with the first reference frameset at the onset of the QRS-wave of the surface ECG as recommended [15]. Values were obtained from a single apical 4-chamber view. For simplicity, in this paper, we refer only to the absolute LA strain value during systole and diastole. LA stiffness was estimated using the E/e’ and LA-reservoir strain ratio, as previously applied to the athlete’s heart [14].

### Statistical analysis

Statistical analysis was performed with MedCalc Statistical Software (version 13.2.0, MedCalc Software bvba, Belgium). A P-value of < 0.05 was considered significant. All continuous variables were tested for normal distribution using the Shapiro-Wilk test. Variables were expressed as mean ± standard deviation if normally distributed, if not, as median ± interquartile range. Comparisons between young and master athletes were performed for continuous variables using parametric (independent samples t-test) or nonparametric (Mann-Whitney test) methods. For proportions, the Chi^2^ or Fisher tests were performed. Univariate and multivariate linear regression analyses were done to identify factors associated with peak e’, LA volume, and LA phasic strains. The following uncorrelated variables (r < 0.8) were included in the analysis: age, gender, duration of the sustained training in years, volume of training/wk, sports disciplines (categorized as endurance, mixed, or other including strength and skill), heart rate, systolic and diastolic BP, LV end-diastolic volume (EDV) indexed, E/A ratio, peak e’, peak a’, and LA volume. The multivariate stepwise model included parameters with a univariate P-value of <0.1.

## Results

### Clinical data

One hundred athletes were eligible for this study, among them 6 subjects were excluded (3 presented high blood pressure, 2 presented cardiomyopathy diseases, and 1 with a history of a Wolff-Parkinson-White syndrome and vasovagal atrial fibrillation). Among the 94 subjects included in the final analysis, 3 patients had mild to moderate asthma and one patient was treated by beta mimetic, 16 athletes were former or current smokers and none reported the use of doping products. None of the athletes included in the study reported paroxysmal atrial fibrillation. The demographic and physical activity data characteristics of study groups are summarized in Table 1. In the overall population, 53 participants were considered as young athletes, and 41 participants were considered as master athletes. The minimal and maximal age range was 18 to 66 years. Diastolic blood pressure was significantly higher in master athletes. Intergroup differences relied on the physical activity showed that the volume of weekly training was higher in younger athletes, but the master athletes had a longer exposure in terms of years of sustained exercise practice. Master athletes were more likely to engage in endurance disciplines whereas young athletes participated rather in mixed sport disciplines. Sixty-two percent of young subjects were professional athletes (33/53), whereas in the master group only one subject was a former professional athlete. The sports categories were established following the classification from the European Association of Preventive Cardiology [16] and detailed in Supporting information (S1 Table).

**Table 1:** Population characteristics.

	Age < 35 yrs (n = 53)	Age ≥ 35 yrs (n = 41)	P
***Demographic data***			
Age, yrs	24 [20.8–27]	43 [41–52.5]	**<0.0001**
Male gender	39 (74%)	36 (88%)	0.12
Height, cm	185 ± 12	175 ± 7	**<0.0001**
Weight, kg	80 [70.8–95]	70 [66–75]	**0.0002**
BMI, kg/m^2^	23.9 [22.3–25.8]	22.7 [21.8–24.6]	0.095
BSA, m^2^	2 [1.88–2.21]	1.83 [1.77–1.9]	**<0.0001**
Heart rate, bpm	57 [50–63.5]	57 [50–63]	0.68
SBP, mmHg	121 ± 9	122 ± 12	0.66
DBP, mmHg	69 ± 8	76 ± 9	**0.0002**
***Physical activity data***			
Volume, hrs/wk	13.1 ± 3.4	9.5 ± 3.1	**<0.0001**
Years of sustained training, yrs	7.2 ± 3.6	13.7 ± 8.9	**0.0003**
Sport discipline			
Endurance	8 (15.1%)	34 (82.9%)	**<0.0001**
Mixed	37 (69.8%)	1 (2.4%)	**<0.0001**
Power	8 (15.1%)	4 (9.8%)	0.54
Skill	0	2 (4.9%)	0.18

Values are expressed as mean ± SD if normally distributed or median and interquartile [25^e^-75^e^ percentile]. Abbreviations: BMI: body mass index, BSA: body surface area, SBP: systolic blood pressure, DBP: diastolic blood pressure.

### Left ventricular analysis

Left ventricular morphological and functional assessment is presented in Table 2. LV linear dimensions were comparable between groups, yet 3D assessment showed higher LV EDV, LV end-systolic volume (ESV), and LV stroke volume indexed to BSA in young athletes. There was no difference in LV mass between groups, however, the mass/volume ratio was slightly higher in master athletes, as well as LV stiffness index. LV EF and peak GLS were preserved and similar between groups. Regarding diastolic function, master athletes had a significantly lower transmitral peak E and a higher peak A, which resulted in a decreased E/A ratio. Similarly, the LV basal e’/a’ ratio was lower in masters resulting from significantly lower peak e’ and higher peak a’, in comparison with young athletes. E/e’ ratio was slightly lower in younger athletes, yet none of the athletes presented an abnormal E/e’ ratio.

**Table 2:** Left ventricular morphology and function.

	Age < 35 yrs (n = 53)	Age ≥ 35 yrs (n = 41)	P
***LV morphological parameters***			
LVIDDi, mm/m^2^	26.4 ± 3	27.5 ± 3.2	0.07
LVIDSi, mm/m^2^	22.9 [15.5–19.3]	22.3 [16.6–19.1]	0.09
IVS, mm	8.7 ± 1.2	8.5 ± 1.5	0.5
PWT, mm	8.4 ± 1.2	8.1 ± 1.4	0.17
LV EDVi, ml/m^2^	93 ± 17	84 ± 14	**0.004**
LV ESVi, ml/m^2^	41 ± 8	36 ± 7	**0.004**
LV SVi, ml/m^2^	52 ± 11	47 ± 8	**0.02**
LV mass i, g/m^2^	99 ± 20	98 ± 19	0.7
LV mass/volume ratio	1.07 ± 0.14	1.17 ± 0.18	**0.002**
LV stiffness	0.029 ± 0.01	0.038 ± 0.011	**0.0002**
***LV functional parameters***			
LV EF, %	55.8 ± 4.7	56.8 ± 4.2	0.32
LV GLS, %	-21.3 ± 3	-21.1 ± 3.1	0.8
Transmitral E-wave, cm/s	76.3 ± 15.9	67.1 ± 14	**0.003**
Transmitral A-wave, cm/s	40.2 ± 8.5	50.1 ± 9.6	**<0.0001**
E/A ratio	1.9 ± 0.3	1.4 ± 0.3	**<0.0001**
TDI e’ basal, cm/s	14.7 ± 2	11.8 ± 2.1	**<0.0001**
TDI a’ basal, cm/s	5.9 ± 1.2	8.2 ± 1.8	**<0.0001**
E/e’ ratio	5.2 ± 1.	5.7 ± 1.2	**0.046**
e’/a’ ratio	2.6 ± 0.8	1.5 ± 0.5	**<0.0001**

Values are expressed as mean ± SD if normally distributed or median and interquartile [25^e^-75^e^ percentile]. Abbreviations: EDV: end-diastolic volume, EF: ejection fraction, ESV: end-systolic volume, GLS: global longitudinal strain, i: indexed, LV: left ventricular, LVIDD: LV internal diastolic dimension, LVIDS: LV internal systolic dimension, IVS: interventricular systolic septum, PWT: posterior wall thickness, SV: stroke volume, TDI: tissue Doppler imaging.

### Left atrial analysis

Table 3 summarizes the LA volumetric and functional parameters. Forty athletes (43%) could be considered having an LA dilatation as described by the standard recommendations established for the general population [13] with a LA volume ≥ 34 ml/m^2^ with a similar distribution between the two groups (22 master and 18 young athletes). Master athletes had a greater mean LA volume indexed, whereas the LA total EF was diminished compared to the young athletes. Regarding LA phasic volumetric assessment, the active phase represented a larger part in the LA global function in masters whereas the LA expansion index and passive EF were lower than the young athletes. Speckle tracking analysis confirmed that LA function in masters was characterized by a significantly greater LASct and reduced LASr and LAScd alongside a greater LA stiffness, in comparison with young athletes (Table 3).

**Table 3:** Left atrial volumetric and functional indexes.

	Age < 35 yrs (n = 53)	Age ≥ 35 yrs (n = 41)	P
***LA volumetric indexes***			
LA volume index, ml/m^2^	30.6 ± 7.8	33.9 ± 7.8	**0.04**
LA total EF, %	60.2 ± 5.4	55.5 ± 6.3	**0.0002**
LA expansion index	1.56 ± 0.35	1.29 ± 0.32	**0.0003**
LA passive EF,%	47.8 [42.1–51.4]	39.3 [31–42]	**<0.0001**
LA active EF, %	25.4 ± 6.4	29.3 ± 7	**0.005**
***LA speckle tracking-derived indexes***			
LASr, %	40.4 ± 6.1	32.7 ± 6.2	**<0.0001**
LAScd, %	33.1 ± 5.4	22.5 ± 6.5	**<0.0001**
LASct, %	9.9 ± 2.3	12 ± 3.8	**0.003**
LA stiffness	0.13 ± 0.03	0.18 ± 0.06	**<0.0001**

Values are expressed as mean ± SD if normally distributed or median and interquartile [25^e^-75^e^ percentile]. Abbreviations: EF: ejection fraction, LA: left atrial, LASr: LA reservoir strain, LAScd: LA conduit strain, LASct: LA contractile strain.

### Variables influencing LV and LA functions

**Fig 1:**
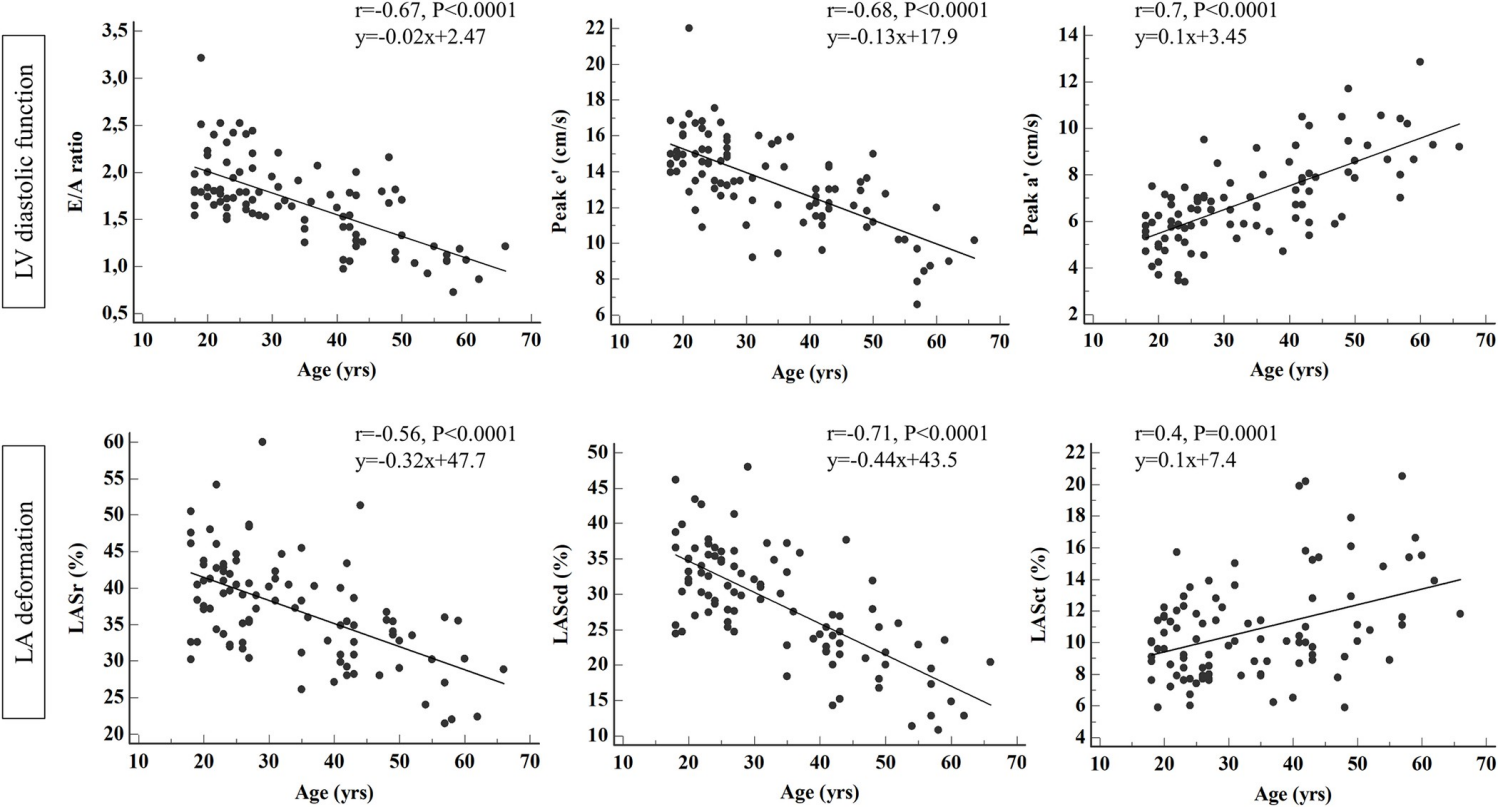
Linear regression analysis between age and left ventricular and atrial functional indices.

As shown in Fig 1, there were highly significant relationships between age and both LV and LA functional parameters. The E/A ratio, peak e’, and LASr and LAScd were negatively correlated with age, whereas positive correlations were found between age and LV peak a’ and LASct. LA and LV stiffness indices were both positively correlated with age (r = 0.56, P < 0.0001; and r = 0.36, P = 0.0004, respectively). In the multivariate regression analysis (Table 4), peak e’ was mainly determined by age with an adjusted R^2^ of 0.48. Age was also predictive of LASr and LAScd which were associated with LV peak e’. LASct has mainly been linked to LV peak a’. Finally, LA enlargement was found to be independently related to the duration of chronic intensive training and LV eccentric remodeling.

**Table 4:** Multiple regression stepwise model analysis.

Dependent variable	Independent variables	β-coefficient	P
Peak e’	Age	-0.13	<0.0001
LA volume indexed	Years of training	0.7	<0.0001
	LV EDV indexed	0.18	<0.0001
LA reservoir strain	Age	-0.21	0.002
	Peak e’	0.81	0.018
LA conduit strain	Age	-0.28	<0.0001
	Peak e’	0.94	0.002
	LV EDV indexed	0.07	0.047
LA contractile strain	Peak a’	0.69	0.0001
	Peak e’	-0.13	0.01

Abbreviations: EDV: end-diastolic volume LA: left atrial, LV: left ventricular, DBP: diastolic blood pressure.

## Discussion

Using echocardiography, the present study demonstrates a strong relationship between age-induced changes in LV diastolic function and LA function in healthy trained athletes. Age was the predominant predictive factor of LV relaxation and LA deformation. Our findings suggest that sustained physical training does not preserve from a shift in the pattern of LV filling as a consequence of the aging process with reduced LV peak e’ coupled with lower LA reservoir and conduit functions and compensated by a greater LA emptying during late diastole.

Unlike the general population for which LA dilatation is considered as a surrogate of the effect of LV diastolic dysfunction and increased LV filling pressure [17], in trained athletes LA enlargement does not reflect atrial dysfunction [3, 9, 18]. LA enlargement is relatively common in athletes and is considered a physiological adaptation to chronic exercise-induced volume overload [5]. In a recent meta-analysis of echocardiographic studies involving over 2400 elite athletes, the authors showed that LA size was 37% higher in athletes as compared to nonathletic controls with a pooled mean LA indexed volume of 31 ml/m^2^ among the athletic population [19]. In accordance, our pooled data showed an averaged LA volume of 32 ml/m^2^ and our multivariate regression analysis confirmed that LA dilatation was associated with the duration of high-intensity training and the LV eccentric remodeling as part of the global cardiac training-induced response. Masters athletes, who presented longer exposure to training were mostly engaged in endurance disciplines (mid-long-distance running, mid-long-distance swimming, and cycling) which are known to lead to the highest level of cardiac adaptations [16, 20]. In addition to a higher degree of LA dilatation, our master athletes exhibited lower LA systolic and early diastolic functional parameters assessed by both 2D volumetric indices and 2D speckle tracking deformation analysis, in comparison to younger athletes. The athlete’s heart is usually characterized by supernormal diastolic function related to a shift in the pattern of ventricular filling period toward early diastole [4, 21]. Several longitudinal observations demonstrated that prolonged intensive training could be associated with a slight and balanced reduction of LA reservoir and contractile strain [22–24]. A recent meta-analysis of 9 echocardiographic studies including a large number of healthy athletes and nonathletic controls showed that LA phasic strain could be slightly lower in athletes compared to controls [10]. The current data in athletic populations, resulting mainly from young athlete cohorts, suggests that exercise-induced atrial remodeling is associated with normal atrial function and compliance [9, 14].

Studies that have analyzed LA strain in master athletes as a marker of LA function/dysfunction are sparse. When comparing mild-aged athletes (43 ± 4 years old) with physiological hypertrophy and patients with hypertrophic cardiomyopathy, Gabrielli et al. [25], highlighted the clinical interest of assessing LA reservoir deformation using General Electric (GE) 2D strain to diagnose early myocardial impairment (LASr was 19% in patients vs 43% in athletes). The authors noticed that LA reservoir strain in athletes was comparable to age-matched sedentary healthy controls. More recently, Hubert et al. [26] examined atrial function using GE 2D strain in male endurance athletes of sixty years old with documented paroxysmal atrial fibrillation compared with their counterparts without documented atrial fibrillation. The authors showed that LA strain was associated with paroxysmal atrial fibrillation and that decreased LA reservoir strain could identify master athletes with maladaptive LA remodeling at risk to develop atrial fibrillation. These observations have an important clinical implication since strenuous endurance exercise is known to be associated with an increased risk of atrial fibrillation in athletes and was correlated with atrial fibrosis in animal models [27]. Therefore, Hubert et al. [26] have suggested that a cut-off LA strain reservoir value of < 39% could be used to distinguish LA dysfunction in master athletes. In comparison, Cuspidi et al. [10] have reported in their meta-analysis, pooling GE LA strain data obtained from predominantly young healthy male athletes, a reference value for normal LA strain reservoir of 37%. Observations made in healthy nonathletic populations showed a normal range of LA reservoir strain between 35.7% and 42.2% using different ultrasound systems [28–30]. These observations highlight the difficulty for a consensus definition of LA early dysfunction in athletes by comparing absolute values of LA strain in the literature. One of the important sources of variability in athlete studies is the heterogeneity of exercise variables between individuals and studies. Moreover, LA strain analysis reliability could be challenging. First, LA strain measurement methodology could vary from one study to another, either in the selection of the apical view (4-chamber, 2-chamber, or an average of the biplane measurement) with significantly higher LA strain obtained in 2-chamber compared to 4-chamber view [29] or in the definition of the zero strain reference that can be set at LV end-diastole or the onset of LA contraction [15]. Furthermore, the intra and inter-vendor variability of speckle tracking derived LA strain measurements should be taken into account when comparing studies with different ultrasound systems [31].

Our study was not designed to evaluate the clinical prognosis of LA strain absolute values. It aimed to explore in an asymptomatic healthy athletic population without known heart disease the effect of aging on atrioventricular functional coupling among highly trained athletes. Our multiparametric regression analysis confirmed that age was the main predictor of LV peak e’ and LA reservoir and conduit functions, independently of the duration and intensity of training. The sport discipline was not found to be predictive of cardiac remodeling, however, mixed and endurance disciplines were predominant in our population. LA deformation analysis by speckle tracking was in accordance with standard LA 2D volumetric indices, both suggesting that LV filling at low atrial pressure in masters requires that LA function shifts towards higher LA contractile contribution. These observations coincide with previous studies suggesting that chronic exposure to intense training does not prevent the gradual age-associated decline in LV diastolic function [32, 33]. Interestingly, LA strain analysis demonstrated close interactions with LV diastolic functional changes, suggesting that LA strain could be useful to explore subtle LV functional changes in athletic populations. Within the limits of a cross-sectional observation, we have no evidence that the functional remodeling observed in master athletes is demonstrative of myocardial LA or LV dysfunction. Although master athletes showed reduced LV and LA early diastolic parameters compared to younger athletes, their averaged LV peak e’ was preserved with low end-diastolic filling pressure. More, LA conduit strain, a reflection of LV suction, was close to data reported among healthy controls where normal LA conduit strain ranged between 23%-25.9% [11, 30]. Although the LA stiffness index was found to be higher in master athletes, it remained below the upper limit of 0.3 described in the literature as a strong predictor of diastolic heart failure, atrial fibrillation, or hypertension [34, 35].

### Study limits

Our study has several limitations. First, our master population was younger compared to other studies that have focused on cardiac adaptations in elderly athletes [26, 33]. However, significant differences in LV diastolic function and LA deformations compared to young athletes were still identified and in this regard, the same conclusions may be expected in older athletes. Secondly, the type of sports practice was heterogeneous in our population. There were significant differences in sports disciplines between younger athletes, mostly engaged in mixed team training, and master athletes mostly engaged in endurance. However, in both groups most sports disciplines involve combined dynamic and static components, and are known to have a high impact on cardiac eccentric remodeling and usually associated with enhanced early diastolic function [21]. More, the sports category was not predictive of left ventricular and atrial function in our study. The sports characteristics were based on unreliable questioning submitting to potential memory bias. Finally, differences in LA function between gender types remain controversial [5, 6], however, the small number of women in this study didn’t allow to bring any conclusion on this subject.

## Conclusion

Healthy aging is known to imply profound changes in LV filling and diastolic function in sedentary populations. Our findings suggested that differences in LA functional remodeling observed between young and master athletes, closely related to LV diastolic function, seemed to be more specific to the aging process than to the effects of sustained exercise practice. Reduction in LA strain has been associated with atrial maladaptive remodeling in both athletic and nonathletic populations [26, 36], however, longitudinal investigations are needed to further determine the clinical significance of LA functional remodeling on cardiovascular outcomes in master athletes.

## Supporting information

S1 Table Description of the sports disciplines in the study population. (DOCX)
