# Supplementary material for: Age impacts left atrial functional remodeling in athletes
Source: PLoS One. 2022 Jul 15;17(7):e0271628. doi: 10.1371/journal.pone.0271628 (PMC9286255; doi:10.1371/journal.pone.0271628)
Supplement: S1 Table — (DOCX) [file pone.0271628.s001.docx]

**Age impacts left atrial functional remodeling in athletes**

Cynthia Cousergue, Eric Saloux, Emmanuel Reboursière^,^ Amelia Rocamora, Paul Milliez, Hervé Normand, Amir Hodzic.

**SUPPLEMENTARY TABLE**

**S1 Table :** Desciption of the sports disciplines in the study population

| **Sports disciplines** | **Age < 35 yrs** | **Age ≥ 35 yrs** |
| --- | --- | --- |
| **Endurance** (overall n=42, 44.7%) | | |
| Mid/long-distance running | 4 | 18 |
| Triathlon | 1 | 15 |
| cycling | 3 | - |
| long-distance swimming | - | 1 |
| **Mixed** (overall n=38, 40.4 %) | | |
| Basketball | 28 | - |
| Handball | 6 | - |
| Ice-hockey | 2 | - |
| Volleyball | 1 | - |
| Waterpolo | - | 1 |
| **Power** (overall n=12, 12.8 %) | | |
| Weightlifting | 3 | 3 |
| Crossfit | 3 | 1 |
| Climbing | 2 | - |
| **Skill** (overall n=2, 2.1 %) | | |
| Karate | - | 1 |
| Auto racing | - | 1 |
